# Time to First Morning Cigarette and Risk of Chronic Obstructive Pulmonary Disease: Smokers in the PLCO Cancer Screening Trial

**DOI:** 10.1371/journal.pone.0125973

**Published:** 2015-05-18

**Authors:** Kristin A. Guertin, Fangyi Gu, Sholom Wacholder, Neal D. Freedman, Orestis A. Panagiotou, Carolyn Reyes-Guzman, Neil E. Caporaso

**Affiliations:** 1 Nutritional Epidemiology Branch, Division of Cancer Epidemiology and Genetics, National Cancer Institute, National Institutes of Health (NIH), Department of Health and Human Services, Bethesda, MD, United States of America; 2 Genetic Epidemiology Branch, Division of Cancer Epidemiology and Genetics, National Cancer Institute, National Institutes of Health (NIH), Department of Health and Human Services, Bethesda, MD, United States of America; 3 Biostatistics Branch, Division of Cancer Epidemiology and Genetics, National Cancer Institute, National Institutes of Health (NIH), Department of Health and Human Services, Bethesda, MD, United States of America; Medical College of Wisconsin, UNITED STATES

## Abstract

**Background:**

Time to first cigarette (TTFC) after waking is an indicator of nicotine dependence. The association between TTFC and chronic obstructive pulmonary disease (COPD), the third leading cause of death in the United States, has not yet been reported.

**Methods:**

We investigated the cross-sectional association between TTFC and prevalent COPD among 6,108 current smokers in the Prostate, Lung, Colorectal, and Ovarian (PLCO) Cancer Screening Trial. COPD was defined as a self-reported diagnosis of emphysema, chronic bronchitis, or both. Current smokers in PLCO reported TTFC, the amount of time they typically waited before smoking their first cigarette of the day after waking, in four categories: ≤5, 6-30, 31-60, or >60 minutes. We used logistic regression models to investigate the association between TTFC and prevalent COPD with adjustments for age, gender, race, education, and smoking (cigarettes/day, years smoked during lifetime, pack-years, age at smoking initiation), and prior lung cancer diagnosis.

**Results:**

COPD was reported by 19% of these 6,108 smokers. Individuals with the shortest TTFC had the greatest risk of COPD; compared to those with the longest TTFC (>60 minutes) the adjusted odds ratios (OR) and 95% confidence intervals (CI) for COPD were 1.48 (95% CI, 1.15-1.91), 1.64 (95% CI, 1.29-2.08), 2.18 (95% CI, 1.65-2.87) for those with TTFC 31-60 minutes, 6-30 minutes, and ≤5 minutes, respectively (P-trend <0.0001). The association between TTFC and emphysema was similar to that for bronchitis, albeit the ORs were slightly stronger for chronic bronchitis; comparing TTFC ≤5 minutes to >60 minutes, the adjusted OR (95% CI) was 2.29 (1.69-3.12) for emphysema and 2.99 (1.95-4.59) for chronic bronchitis.

**Conclusions:**

Current smokers with shorter TTFC have increased risk of COPD compared to those with longer TTFC, even after comprehensive adjustment for established smoking covariates. Future epidemiologic studies, including prospective designs, should incorporate TTFC to better assess disease risk and evaluate the potential utility of TTFC as a COPD screening tool for smokers in the clinical setting.

## Introduction

It is estimated that smoking is the primary causal factor for 80% of deaths from chronic obstructive pulmonary disease (COPD) [1], the third leading cause of death in the United States [2]. COPD leads to increased morbidity, mortality, and health care costs [2]. The risk of COPD can be reduced by never smoking, and smoking cessation may alleviate symptoms and slow progression of the disease [3–5]. Nevertheless, approximately 20% of the U.S. population smokes [6]. Among those with COPD, 30–40% persist in smoking after diagnosis [7, 8]. More detailed characterization of smoking behaviors may help to identify those at greatest risk of disease for additional screening and intervention with the goal of reducing COPD incidence and mortality.

Time to first cigarette (TTFC) is a measure of the time from waking until an individual smokes their first cigarette of the day. TTFC is a single indicator of nicotine dependency which could plausibly contribute to quantifying disease risk, as it has for lung cancer [9, 10]; however, the association between TTFC and COPD risk has not yet been investigated.

Smoking behavior and addiction are assessed by the Fagerström Test for Nicotine Dependence (FTND), a six-item questionnaire that queries about smoking behaviors, including the number of cigarettes smoked per day and difficulty in refraining from smoking in certain situations [11]. TTFC, the first item of FTND, reflects nicotine dependence and is related to the ability to quit [12]. Most epidemiologic studies take into account cigarettes per day and duration, but there is strong evidence that TTFC provides additional information which may be important in assessing smoking-related disease risk.

For example, in a recent study among a nationally representative sample in the US, individuals with shorter TTFC had greater tobacco carcinogen levels (creatinine-adjusted urinary 4-(methylnitrosamino)-1-(3-pyridyl)-1-butanol (NNAL)), even after adjustment for other smoking factors [13]. TTFC is also predictive of both plasma and urinary cotinine levels, even after adjustment for the number of cigarettes smoked per day [14]. Recently, we reported that TTFC differentiates lung cancer risk and provides information beyond usual smoking metrics [9]. Furthermore, a recent Canadian study demonstrated that current smokers with COPD had greater odds of high nicotine addiction, as measured by the total FTND score compared to smokers without COPD [7]. This suggests that TTFC may help to quantify disease risk, but no study has yet reported the association between TTFC and COPD.

Lung function testing is the gold standard for COPD diagnosis but clinical guidelines [15] advise against screening asymptomatic patients for COPD with due to the associated costs [16]. Thus, asymptomatic smokers may easily be missed; a single question, such as TTFC, which could be used as a screening tool for COPD risk in smokers would be cost-effective in terms of time and economic and healthcare resources.

Therefore, we evaluated the association between TTFC and COPD in a cross-sectional sample from the Prostate, Lung, Colorectal, and Ovarian (PLCO) Cancer Screening Trial [17, 18].

## Methods

### Study population

Our study is a cross-sectional analysis within current smokers in the PLCO Cancer Screening Trial. PLCO, described in detail elsewhere [17, 18], is a large multicenter randomized screening trial for four cancer sites. Briefly, PLCO randomized over 150,000 men and women aged 55–74 in ten screening centers across the US between 1993 and 2001. PLCO eligibility criteria included no history of cancers of the prostate, lung, colorectum, or ovaries.

PLCO administered two questionnaires which queried about health and lifestyle factors, including smoking and lung disease. The first questionnaire was administered at PLCO baseline, between 1993–2001, with the date of administration dependent upon a participant’s date of randomization. The second questionnaire was administered during PLCO follow-up in 2006 to all participants; average duration between PLCO baseline and follow-up questionnaire was 8.7 (SD 1.8) years.

Eligibility criteria for our study included non-missing data for both TTFC and self-reported COPD. Among PLCO participants, 6,162 current smokers reported both TTFC and COPD information. After excluding 54 subjects with missing data for covariates included in our statistical models, our analyses were comprised of 6,108 participants.

The PLCO trial was approved by the institutional review boards of the US National Cancer Institute and the 10 screening centers, including the University of Colorado, Georgetown University, Pacific Health Research & Education Institute, Henry Ford Health System, University of Minnesota, Washington University, University of Pittsburgh, University of Utah, Marshfield Clinic Research Foundation, and the University of Alabama at Birmingham. All participants provided written informed consent.

### Exposure and outcome assessment

TTFC information was collected on the PLCO follow-up questionnaire in 2006; since TTFC reflects current smoking habits, only current smokers were queried about TTFC. The question was “How soon after you wake up do you usually smoke your first cigarette of the day?” Participants selected their response from the following four categories: within 5, 6–30, 30–60, or >60 minutes of waking.

We defined COPD cases as participants who self-reported a diagnosis of emphysema (on PLCO baseline questionnaire, follow-up questionnaire in 2006, or both), chronic bronchitis (on PLCO baseline questionnaire), or both. The PLCO baseline questionnaire queried specifically about a doctor’s diagnosis of emphysema and chronic bronchitis, whereas the follow-up questionnaire did not query about chronic bronchitis and did not specify whether the diagnosis was by a doctor. References to baseline and follow-up in the manuscript refer to the larger PLCO study design [17, 18]. Covariates were based on data collected at the time of the TTFC query on the follow-up questionnaire.

### Statistical methods

We used logistic regression models to estimate the COPD odds ratios for each TTFC category, with TTFC >60 minutes as the referent group. Models were adjusted for demographic variables including age at PLCO questionnaire (continuous), gender, race (white non-Hispanic, other), and education (≤high school, some college, ≥college). We also adjusted for smoking-related variables to assess the additional risk that TTFC conferred; these variables included cigarettes per day smoked (≤5 cigarettes per day, 6–19 cigarettes per day, 1 pack per day, >1 pack per day), years smoked during a lifetime (continuous), pack-years (continuous), age at smoking initiation (<17, ≥17 years), and prior diagnosis of lung cancer (yes/no). Further adjustment for study center (10 locations) did not appreciably change results (Table A in S1 File), thus to avoid over-fitting the model, study center is not included in the main models. We also explored the possibility that lung cancer diagnoses prior to the follow-up questionnaire biased our estimates by excluding these individuals from our models in a sensitivity analysis. Lastly, we conducted a sensitivity analysis among only participants with complete follow-up questionnaire data (n = 5,530), without allowing for any substitution of baseline data for covariates. In a small number of instances of missing follow-up data for covariates, individual values were imputed based on baseline values; such substitutions occurred for race, age at smoking initiation, cigarettes per day, pack-years, and smoking duration (years smoked).

We investigated several pre-specified subgroup analyses, including stratification by gender and race. Additionally, in separate models we considered the association between TTFC and chronic bronchitis, as well as TTFC and emphysema. We also evaluated the effect of adjusting models of emphysema for chronic bronchitis, and adjusting models of chronic bronchitis for emphysema. Lastly, since emphysema was reported at both PLCO baseline and PLCO follow-up, we further examined the concordance of these reports; we also conducted a sensitivity analyses by comparing the results using emphysema reported at baseline and at follow-up.

SAS 9.1.3 (SAS Institute, Cary, NC) was used for all analyses.

## Results

### Participant characteristics

In total, 6,108 current smokers in PLCO met our criteria for inclusion in this study (Table 1). Over half (62%) of smokers reported smoking their first morning cigarette within 30 minutes of waking, with 16% reporting smoking within 5 minutes of waking. Given that the larger PLCO study was comprised mostly of non-Hispanic whites, our study sample of current smokers is also largely homogenous in terms of race; these current smokers were comprised mostly of non-Hispanic whites (89%). The mean age at baseline was 69, and level of education varied widely; approximately one-third of participants achieved no greater than a high school diploma, and one-quarter had at least a college degree. On average, individuals reported a smoking history of 50 years and accumulated 44 pack-years; at baseline most (80%) of smokers reported a usual smoking intensity of at least 6 cigarettes/day. Most of these current smokers (63%) initiated smoking at the age of 17 or later. A small portion of current smokers (n = 77, 1%) reported a history of lung cancer.

**Table 1:** Characteristics of Current Smokers in the PLCO Study, by COPD Status.

Characteristic	Non-Cases	COPD Cases	Total
**N**	4972	1136	6108
**Age (years), mean ± SD**	69 (5)	70 (5)	69 (5)
**Male, N (%)**	2533 (51)	561 (49)	3094 (51)
**Non-Hispanic white, N (%)**	4364 (88)	1052 (93)	5416 (89)
**Education, N (%)**			
≤High school	1615 (33)	445 (39)	2060 (34)
Some college	2041 (41)	456 (40)	2497 (41)
College graduate	1316 (26)	235 (21)	1551 (25)
**Time to first cigarette (minutes), N (%)**			
>60	1048 (21)	136 (12)	990 (16)
31–60	957 (19)	195 (17)	2782 (46)
6–30	2236 (45)	546 (48)	2782 (46)
≤5	731 (15)	259 (23)	990 (16)
**Cigarettes per day, N (%)**			
≤5	1055 (21)	165 (15)	1220 (20)
6–19	1787 (36)	363 (32)	2150 (35)
20	1442 (29)	379 (33)	1821 (30)
>20	688 (14)	229 (20)	917 (15)
**Pack-years (packs/day × year), mean ± SD**	43 (23)	50 (25)	44 (24)
**Smoking duration (years), mean, ± SD**	50 (8)	52 (7)	50 (8)
**Age at smoking initiation <17 years, N (%)**	1730 (35)	531 (47)	2261 (37)
**Prior lung cancer diagnosis, N (%)** ^**a**^	44 (1)	33 (3)	77 (1)
**Emphysema, N (%)** ^**b**^	0 (0)	882 (78)	882 (14)
**Chronic bronchitis, N (%)** ^**b**^	0 (0)	455 (40)	455 (7)

COPD cases responded affirmatively to question(s) regarding diagnosis of emphysema (on study baseline questionnaire, follow-up questionnaire in 2006, or both), chronic bronchitis (on study baseline questionnaire), or both. Baseline data were substituted for missing follow-up questionnaire data for the following variables (number of participants) for race (218), age at smoking initiation (108), pack-years (299), cigarettes per day (65), smoking duration (262). PLCO, Prostate, Lung, Colorectal, and Ovarian (PLCO) Cancer Screening Trial. Some values do not sum to 100 due to missing data.

^a^ Report prior to PLCO follow-up questionnaire in 2006

^b^ Self-report of emphysema and chronic bronchitis was not mutually exclusive; 201 participants reported a diagnosis of both emphysema and chronic bronchitis, thus the sum of reports of emphysema and chronic bronchitis are greater than the total 1,136 COPD cases.

Nineteen percent (n = 1,136) of current smokers self-reported diagnosis of emphysema, chronic bronchitis, or both, thereby meeting our definition of COPD. Among COPD cases, 78% reported emphysema and 40% reported bronchitis; reporting of emphysema and chronic bronchitis were not mutually exclusive, and 18% of COPD cases reported both diagnoses. COPD diagnoses were approximately evenly distributed by gender. Compared to non-cases, individuals with COPD had lower education and smoked more cigarettes per day.

### Risk of COPD, emphysema, and bronchitis

The risk of COPD was higher among smokers with shorter TTFC (Table 2). Compared to smokers with longer TTFC (>60 minutes), the multivariate adjusted ORs (95% CI) for COPD were 1.48 (1.15–1.91), 1.64 (1.29–2.08), 2.18 (1.65–2.87) for those with TTFC 31–60 minutes, 6–30 minutes, and ≤5 minutes, respectively (P-trend <0.0001). Subgroup analyses by gender showed similar trends (Table 2). In gender-stratified analyses, ORs between TTFC and COPD, chronic bronchitis, and emphysema were all statistically significant in both men and women. The point estimate for the risk of COPD conferred by shortened TTFC was higher among females, although the interaction was not statistically significant (P-value = 0.13 for TTFC*gender interaction term). Importantly, the trend of increased risk of COPD with shortened TTFC was consistent by gender and disease subtype.

**Table 2:** Adjusted Odds Ratio for COPD according to Time to First Cigarette (TTFC) among current smokers in PLCO.

			Time to First Cigarette (minutes)				P-Values	
Disease Endpoint	No. Cases	No. Controls	>60 (Ref.)	31–60	6–30	≤5	P-Trend	TTFC*gender
**COPD**								
All	1136	4972	1.00	1.48 (1.15–1.91)	1.64 (1.29–2.08)	2.18 (1.65–2.87)	<0.01	0.13
Male	561	2533	1.00	1.42 (0.99–2.02)	1.38 (0.99–1.93)	2.05 (1.39–3.01)	<0.01	
Female	575	2439	1.00	1.53 (1.05–2.20)	1.94 (1.38–2.72)	2.32 (1.56–3.45)	<0.01	
**Emphysema** ^**a**^								
All	882	5224	1.00	1.46 (1.10–1.95)	1.70 (1.30–2.22)	2.29 (1.69–3.12)	<0.01	0.58
Male	484	2608	1.00	1.36 (0.93–1.99)	1.45 (1.01–2.06)	2.17 (1.44–3.27)	<0.01	
Female	398	2616	1.00	1.55 (1.01–2.40)	2.04 (1.37–3.05)	2.45 (1.54–3.91)	<0.01	
**Chronic bronchitis** ^b^								
All	455	5615	1.00	2.05 (1.37–3.07)	1.85 (1.25–2.73)	2.99 (1.95–4.59)	<0.01	0.26
Male	163	2915	1.00	2.38 (1.22–4.64)	1.60 (0.83–3.09)	3.08 (1.51–6.26)	0.02	
Female	292	2700	1.00	1.83 (1.10–3.04)	2.01 (1.23–3.25)	2.95 (1.72–5.07)	<0.01	

COPD cases responded affirmatively to question(s) regarding diagnosis of emphysema (on study baseline questionnaire, follow-up questionnaire in 2006, or both), chronic bronchitis (on study baseline questionnaire), or both. ORs and 95% CIs were determined using logistic regression with adjustments for age, gender (except in gender-stratified analyses), race, education, cigarettes/day, years smoked during lifetime, pack-years, age at smoking initiation, and lung cancer diagnosis prior to follow-up questionnaire. TTFC was categorical (>60 minutes as reference) with the exception of P-trend assessment where TTFC was treated as an ordinal variable.

PLCO, Prostate, Lung, Colorectal, and Ovarian (PLCO) Cancer Screening Trial. P-value for interaction between TTFC*gender interaction term. Significant P-values (P<0.05) in bold.

^a^ N = 6,106; 2 participants were excluded due to missing data on emphysema endpoint.

^b^ N = 6,070; 38 participants were excluded due to missing data on bronchitis endpoint.

All TTFC categories showed an increased risk of disease (COPD, chronic bronchitis, and emphysema separately) compared to those with TTFC>60 minutes (P <0.05). The point estimate of COPD risk conferred by shortened TTFC was of greater magnitude for chronic bronchitis compared to emphysema, particularly among those with TTFC ≤5 minutes (ORs 2.29 and 2.99 for emphysema and chronic bronchitis, respectively).

**Fig 1:**
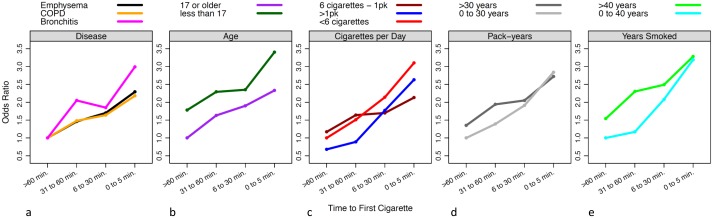
Time to first cigarette (TTFC) upon waking and risk of chronic obstructive pulmonary disease (COPD) among current smokers. Odds Ratios for each category of TTFC (≤5, 6–30, and 31–60 minutes, compared to the reference group of smokers with TTFC >60 minutes) were calculated by logistic regression, adjusted for age, gender (except in gender-stratified analyses), race, education, cigarettes/day, years smoked during lifetime, pack-years, age at smoking initiation, and lung cancer diagnosis prior to follow-up questionnaire. (A) OR for COPD, emphysema, and chronic bronchitis. (B) OR for COPD by age at smoking initiation. (C) OR for COPD by typical number of cigarettes smoked per day. (D) OR for COPD by pack-years. (E) OR for COPD by total smoking duration in years. Categories of smoking covariates were collapsed for visual representation of results; refer to Table 2 for further details.

TTFC was strongly associated with COPD, even when participants were stratified by other smoking covariates. The association persisted across stratum of smoking duration (Fig 1). For example, risk of COPD increased as TTFC shortened among both those who smoked for 40 years or less and those who smoked for 40 y or more, for whom the ORs (95% CI) were 1.17 (0.43–3.18), 2.08 (0.92–4.69) and 3.19 (1.19–8.53) for TTFC 31–60, 6–30, and <5 minutes compared to TTFC >60 minutes, respectively (Fig 1 and Table B in S1 File). Similar patterns were observed across stratum of other examined smoking phenotypes including cigarettes per day, pack-years, years smoked, and age at smoking initiation.

### Sensitivity and subgroup analyses

The association between TTFC and COPD was consistent in a sensitivity analysis limited to participants without a prior diagnosis of lung cancer (yes/no) (Table C in S1 File) and, in a separate model, after adjusting for such diagnoses (n = 33 cases; n = 44 controls). In models limited to participants with complete (non-imputed) follow-up data (Table D and Table E in S1 File), results were similar to our main findings. In models which considered emphysema and chronic bronchitis separately, adjusting for the other condition attenuated the association slightly but statistical significance was retained (Table F in S1 File). The association between TTFC and the risk of COPD in non-Hispanic whites (Table G in S1 File) was almost identical that for the overall sample of current smokers. Among African Americans, those with shorter TTFC had the greatest risk of COPD and emphysema, compared to those with longest TTFC (Table H in S1 File). Given the small number of African Americans in this sample (n = 360), risk estimates for chronic bronchitis could not be produced.

Since emphysema was reported at both PLCO baseline and PLCO follow-up, we explored the concordance of these reports. Almost half (n = 333) of the total 882 emphysema cases were reported at baseline. Concordance between reports of emphysema at baseline and follow-up questionnaires was high; among the 882 emphysema cases in our analyses, 26% (n = 229) reported emphysema on both baseline and follow-up questionnaires, and 62% (n = 545) reported emphysema at follow-up but not at baseline. Discordant responses were recorded for 9% (n = 80) of cases; these participants reported emphysema on the first encounter but not the second. Three percent of emphysema cases were missing emphysema data on one questionnaire (n = 4 missing baseline emphysema data, n = 24 missing follow-up emphysema data). In logistic regression models, we observed similar associations between TTFC and emphysema reported at baseline and at follow-up. Compared to TTFC >60 minutes, the multivariate adjusted ORs (95% CI) for emphysema at the baseline questionnaire were 1.49 (0.95–2.36), 1.50 (0.98–2.31), and 2.19 (1.36–3.54) for TTFC 31–60 minutes, 6–30 minutes, and ≤5 minutes, respectively; at the follow-up questionnaire the multivariate adjusted ORs for emphysema for the same comparisons were 1.43 (1.05–1.94), 1.83 (1.38–2.43), and 2.50 (1.80–3.47), respectively.

## Discussion

Among current smokers in PLCO, shorter TTFC (and thus, greater nicotine dependence) was strongly associated with increased risk of COPD. The association was statistically significant even after adjustment for smoking covariates most commonly used in epidemiologic studies, including cigarettes per day, years smoked during a lifetime, and pack-years.

There are several plausible mechanisms by which shorter TTFC confers a greater risk of COPD. For example, highly dependent smokers inhale more deeply [19], which leads to a higher dose of cigarette-associated carcinogens [20–22]. There may also be other smoking differences between smokers with and without COPD that are not captured by established smoking metrics but are associated with TTFC; these factors include how much of the cigarette is smoked before it is extinguished and how long smoke is retained in the lungs after inhalation and prior to exhalation.

As is evident from the ORs in Fig 1, chronic bronchitis was more strongly influenced by TTFC than was emphysema. There are a few possible explanations for our finding of a stronger association for chronic bronchitis, compared to emphysema. First, TTFC may be more strongly associated with chronic bronchitis. Secondly, reporting for chronic bronchitis may be more accurate. Finally, chance must be considered since the confidence intervals do overlap.

TTFC is a measure of nicotine addiction, but it has been largely absent from epidemiologic investigations of lung disease, including a systematic review of smoking and COPD [23]. To our knowledge, ours is the first to investigate the association between TTFC and COPD in a substantial sample from a large representative cohort. Consistent with our findings are reports that shorter TTFC is associated with family history of COPD [24] and increased risk of lung [9, 10, 25, 26] and supraglottic cancers [27].

There have been several reports of associations between the FTND score, from which TTFC is derived, and respiratory endpoints. Higher FTND scores, which indicate greater addiction to nicotine and smoking [11], are associated with greater risk of COPD [7, 28], although the association may vary by sex [7]. While one study using computed tomography (CT) imaging found that FTND score was inversely correlated with CT-defined emphysema and COPD severity, the authors conceded that these findings may be largely due to selection bias and other biological processes which may lead to increased lung density in smokers [29]. Smokers with COPD have higher FTND scores, which reflects greater dependency on nicotine, compared to both smokers without COPD [8] and to individuals who quit smoking after COPD diagnosis [28, 30]. Higher FTND scores were also associated with emergency-room admission for smoking-related conditions, compared to admission for other conditions [31], and with more negative symptoms among smokers engaged in cessation efforts [32]. These data, along with our findings, suggest that TTFC is an important predictor of lung disease, but further evaluation is needed. In particular, longitudinal studies are needed to calculate the lifetime risk of COPD and lung cancer in absolute terms and to increase the translational value of these results.

Our findings should be interpreted with a few caveats. COPD cases in this study were identified by self-reports of diagnosis of emphysema, chronic bronchitis, or both; these cases are highly likely to represent truly diseased individuals, but a degree of non-differential misclassification may be present [33]. TTFC was only assessed once (six years post-baseline), but this assessment was at an age when dependency is likely to be highly stable; study participants had, on average, smoked for 50 years and over one-third of our sample initiated this lifelong behavior prior to age 17. The effects of misclassification of COPD and TTFC on our estimates, if any, would be to attenuate the true association, but we detected a robust significant increased risk of COPD with shortened TTFC. PLCO questionnaires queried both emphysema and chronic bronchitis, but did not specifically use the term COPD; future studies should ask participants about emphysema, chronic bronchitis, and COPD, which may capture a greater portion of individuals with disease. Although we included participants in the screening and control arms of PLCO there was no evidence of differential detection, with COPD identified in 19% of participants in each arm.

We cannot exclude the possibility of selection bias in our study. Our study included current smokers in PLCO who were alive, free of lung cancer and completed the follow-up questionnaire which included TTFC. Thus, some highly addicted smokers with the shortest TTFC would have been excluded, biasing findings toward the null. Conversely, individuals who persist in smoking following a diagnosis of COPD are likely more addicted compared to individuals who quit post-diagnosis [19, 30]; current smokers who report COPD at the follow-up questionnaire may be enriched with individuals with shorter TTFC, and we may therefore overestimate the association between TTFC and COPD. These two biases are in opposite directions and the overall effect is likely to be of small magnitude; our risk estimates should approximate the true association. Evidence from studies of other smoking-related diseases suggest that former smokers will exhibit a similar pattern of disease risk in relation to TTFC, as we recently reported for lung cancer [9].

In summary, TTFC was significantly associated with COPD after simultaneous adjustment for established smoking factors and COPD risk factors; current smokers with shorter TTFC had greater risk of COPD compared to those with longer TTFC (>60 minutes). Although current smokers are at high risk for developing COPD, not all smokers develop COPD [8]. Identification of subgroups of smokers at greatest risk of COPD may help to target clinical screening for this disease, to reduce the number of undiagnosed cases, and thus allow for targeted interventions which may slow the disease progression. Our data suggest that TTFC may provide additional useful information about COPD risk. However, additional studies set in other populations, including former smokers, are needed to confirm our findings. Large prospective studies that define COPD using spirometry are particularly needed.

## Supporting Information

S1 File Table A in S1 File, Adjusted Odds Ratio (95% CI) for COPD according to TTFC among current smokers in PLCO, with further adjustment for study center. Table B in S1 File, Adjusted Odds Ratio (95% CI) for COPD according to TTFC among current smokers in PLCO in strata defined by traditional smoking covariates. Table C in S1 File, Adjusted Odds Ratio (95% CI) for COPD according to TTFC among current smokers in PLCO, excluding participants with lung cancer diagnosis prior to follow-up questionnaire. Table D in S1 File, Adjusted Odds Ratio (95% CI) for COPD according to TTFC among current smokers in PLCO, limited to participants with complete data on cigarettes per day at follow-up questionnaire. Table E in S1 File, Adjusted Odds Ratio (95% CI) for COPD according to TTFC among current smokers in PLCO, excluding participants missing follow-up questionnaire data for any covariates. Table F in S1 File, Adjusted Odds Ratio (95% CI) for emphysema and chronic bronchitis among current smokers in PLCO, with mutual adjustment for emphysema/chronic bronchitis. Table G in S1 File, Adjusted Odds Ratio (95% CI) for COPD among current smokers in PLCO, among Non-Hispanic Whites. Table H in S1 File, Adjusted Odds Ratio (95% CI) for COPD among current smokers in PLCO, among African Americans.
(DOC)
