# Supplementary material for: Time to First Morning Cigarette and Risk of Chronic Obstructive Pulmonary Disease: Smokers in the PLCO Cancer Screening Trial
Source: PLoS One. 2015 May 18;10(5):e0125973. doi: 10.1371/journal.pone.0125973 (PMC4436174; doi:10.1371/journal.pone.0125973)
Supplement: S1 File — Table A in S1 File, Adjusted Odds Ratio (95% CI) for COPD according to TTFC among current smokers in PLCO, with further adjustment for study center. Table B in S1 File, Adjusted Odds Ratio (95% CI) for COPD according to TTFC among current smokers in PLCO in strata defined by traditional smoking covariates. Table C in S1 File, Adjusted Odds Ratio (95% CI) for COPD according to TTFC among current smokers in PLCO, excluding participants with lung cancer diagnosis prior to follow-up questionnaire. Table D in S1 File, Adjusted Odds Ratio (95% CI) for COPD according to TTFC among current smokers in PLCO, limited to participants with complete data on cigarettes per day at follow-up questionnaire. Table E in S1 File, Adjusted Odds Ratio (95% CI) for COPD according to TTFC among current smokers in PLCO, excluding participants missing follow-up questionnaire data for any covariates. Table F in S1 File, Adjusted Odds Ratio (95% CI) for emphysema and chronic bronchitis among current smokers in PLCO, with mutual adjustment for emphysema/chronic bronchitis. Table G in S1 File, Adjusted Odds Ratio (95% CI) for COPD among current smokers in PLCO, among Non-Hispanic Whites. Table H in S1 File, Adjusted Odds Ratio (95% CI) for COPD among current smokers in PLCO, among African Americans. (DOC) [file pone.0125973.s001.doc]

**Online-Only Supplement**

Table A. Adjusted Odds Ratio (95% CI) for COPD according to TTFC among current smokers in PLCO, with further adjustment for study center

Table B. Adjusted Odds Ratio (95% CI) for COPD according to TTFC among current smokers in PLCO in strata defined by traditional smoking covariates

Table C. Adjusted Odds Ratio (95% CI) for COPD according to TTFC among current smokers in PLCO, excluding participants with lung cancer diagnosis prior to follow-up questionnaire

Table D. Adjusted Odds Ratio (95% CI) for COPD according to TTFC among current smokers in PLCO, limited to participants with complete data on cigarettes per day at follow-up questionnaire

Table E. Adjusted Odds Ratio (95% CI) for COPD according to TTFC among current smokers in PLCO, excluding participants missing follow-up questionnaire data for any covariates

Table F. Adjusted Odds Ratio (95% CI) for emphysema and chronic bronchitis among current smokers in PLCO, with mutual adjustment for emphysema/chronic bronchitis

Table G. Adjusted Odds Ratio (95% CI) for COPD among current smokers in PLCO, among Non-Hispanic Whites

Table H. Adjusted Odds Ratio (95% CI) for COPD among current smokers in PLCO, among African Americans

**Table A. Adjusted Odds Ratio (95% CI) for COPD according to TTFC among current smokers in PLCO, with further adjustment for study center**

|  | **No. Cases/**  **No. Controls** | **Time to First Cigarette (minutes)** | | | | **P-Trend** |
| --- | --- | --- | --- | --- | --- | --- |
| **Disease Endpoint** |  | **>60 (Ref.)** | **31-60** | **6-30** | **≤5** |  |
| COPD |  |  |  |  |  |  |
| All | 1136/4972 | 1.00 | 1.47 (1.14-1.90) | 1.62 (1.28-2.07) | 2.17 (1.64-2.86) | <0.0001 |
| Male | 561/2533 | 1.00 | 1.36 (0.95-1.94) | 1.34 (0.96-1.88) | 2.03 (1.38-2.99) | 0.0010 |
| Female | 575/2439 | 1.00 | 1.52 (1.05-2.19) | 1.92 (1.37-2.71) | 2.30 (1.54-3.43) | <0.0001 |
| Emphysema |  |  |  |  |  |  |
| All | 882/5224 | 1.00 | 1.44 (1.08-1.91) | 1.67 (1.28-2.19) | 2.23 (1.67-3.10) | <0.0001 |
| Male | 484/2608 | 1.00 | 1.31 (0.90-1.92) | 1.40 (0.98-2.00) | 2.14 (1.42-3.22) | 0.0005 |
| Female | 398/2616 | 1.00 | 1.54 (1.00-2.38) | 2.04 (1.36-3.06) | 2.48 (1.55-3.96) | <0.0001 |
| Chronic bronchitis |  |  |  |  |  |  |
| All | 455/5615 | 1.00 | 2.05 (1.37-3.07) | 1.86 (1.26-2.74) | 3.00 (1.95-4.61) | <0.0001 |
| Male | 163/2915 | 1.00 | 2.23 (1.14-4.36) | 1.56 (0.80-3.02) | 3.11 (1.53-6.35) | 0.0127 |
| Female | 292/2700 | 1.00 | 1.79 (1.07-2.99) | 1.99 (1.23-3.23) | 2.90 (1.68-4.99) | 0.0002 |

COPD cases responded affirmatively to question(s) regarding diagnosis of emphysema (on study baseline questionnaire, follow-up questionnaire in 2006, or both), chronic bronchitis (on study baseline questionnaire), or both. ORs and 95% CIs determined using logistic regression with adjustments for age, gender (except in gender-stratified analyses), race, education, cigarettes/day, years smoked during lifetime, pack-years, age at smoking initiation, and lung cancer diagnosis prior to follow-up questionnaire. TTFC was categorical (>60 minutes as reference) with the exception of P-trend assessment where TTFC was treated as an ordinal variable. PLCO, Prostate, Lung, Colorectal, and Ovarian (PLCO) Cancer Screening Trial.

**Table B. Adjusted Odds Ratio (95% CI) for COPD according to TTFC among current smokers in PLCO in strata defined by traditional smoking covariates**

|  |  | **Time to First Cigarette (minutes)** | | | |
| --- | --- | --- | --- | --- | --- |
| **Disease Endpoint** | **No. Cases/**  **No. Controls** | **>60 (Ref.)** | **31-60** | **6-30** | **≤5** |
| COPD | 1136/4972 | 1.00 (Ref.) | 1.48 (1.15-1.91) | 1.64 (1.29-2.08) | 2.18 (1.65-2.87) |
| Emphysema | 575/2439 | 1.00 (Ref.) | 1.46 (1.10-1.95) | 1.70 (1.30-2.22) | 2.29 (1.69-3.12) |
| Chronic bronchitis | 561/2533 | 1.00 (Ref.) | 2.05 (1.37-3.07) | 1.85 (1.25-2.73) | 2.99 (1.94-4.59) |
| COPD, by years smoked during lifetime ^a^ |  |  |  |  |  |
| ≤40 years | 52/433 | 1.00 (Ref.) | 1.17 (0.43-3.18) | 2.08 (0.92-4.69) | 3.19 (1.19-8.53) |
| >40 years | 1087/4557 | 1.54 (0.76-3.12) | 2.30 (1.14-4.67) | 2.49 (1.24-5.02) | 3.28 (1.61-6.70) |
| COPD, by cigarettes smoked per day ^b^ |  |  |  |  |  |
| <6 cigarettes | 170/1086 | 1.00 (Ref.) | 1.51 (0.98-2.14) | 2.14 (1.42-3.23) | 3.10 (1.24-7.75) |
| 6 cigarettes-1 pack | 743/3239 | 1.17 (0.78-1.76) | 1.64 (1.16-2. 32) | 1.70 (1.23-2.37) | 2.13 (1.46-3.1) |
| >1 pack | 229/691 | 0.68 (0.08-5.63) | 0.89 (0.37-2.15) | 1.77 (1.02-3.08) | 2.63 (1.46-4.72) |
| COPD, by pack-years ^c^ |  |  |  |  |  |
| 0-30 pack-years | 186/1251 | 1.00 (Ref.) | 1.39 (0.92-2.10) | 1.91 (1.29-2.81) | 2.84 (1.28-6.33) |
| >30 pack-years | 953/3736 | 1.35 (0.77-2.36) | 1.94 (1.14-3.28) | 2.05 (1.23-3.41) | 2.72 (1.60-4.62) |
| COPD, by age at smoking initiation ^d^ |  |  |  |  |  |
| <17 years | 534/1767 | 1.78 (1.22-2.62) | 2.29 (1.58-3.34) | 2.35 (1.70-3.27) | 3.40 (2.37-4.89) |
| ≥17 years | 608/3253 | 1.00 (Ref.) | 1.63 (1.18-2.26) | 1.90 (1.42-2.56) | 2.33 (1.63-3.33) |

COPD cases responded affirmatively to question(s) regarding diagnosis of emphysema (on study baseline questionnaire, follow-up questionnaire in 2006, or both), chronic bronchitis (on study baseline questionnaire), or both. ORs and 95% CIs determined using logistic regression with adjustments for age, gender, race, education, cigarettes/day, years smoked during lifetime, pack-years, age at smoking initiation, and lung cancer diagnosis prior to follow-up questionnaire. TTFC was categorical and TTFC>60 minutes was the reference group, unless otherwise noted. PLCO, Prostate, Lung, Colorectal, and Ovarian (PLCO) Cancer Screening Trial.

^a^ TTFC incorporated into combined categories which encompassed both TTFC and years smoked during lifetime; participants with TTFC>60 minutes who smoked cigarettes for ≤40 years were the referent group. 33 participants were missing data on years smoked during lifetime.

^b^ TTFC incorporated into combined categories which encompassed both TTFC and cigarettes smoked per day; participants with TTFC>60 minutes who smoked <6 cigarettes/day were the referent group. 4 participants were missing data on the number of cigarettes smoked per day.

^c^ TTFC incorporated into combined categories which encompassed both TTFC and pack-years; participants with TTFC>60 minutes and 0-30 pack-years were the referent group. 36 participants were missing data on the number of cigarette pack-years.

^d^ TTFC incorporated into combined categories which encompassed both TTFC and age at smoking initiation; participants with TTFC>60 minutes who initiated smoking cigarettes at ≥17 years of age were the referent group.

**Table C. Adjusted Odds Ratio (95% CI) for COPD according to TTFC among current smokers in PLCO, excluding participants with lung cancer diagnosis prior to follow-up questionnaire**

|  | **No. Cases/**  **No. Controls** | **Time to First Cigarette (minutes)** | | | | **P-value for**  **Trend** |
| --- | --- | --- | --- | --- | --- | --- |
| **Disease Endpoint** |  | **>60 (Ref.)** | **31-60** | **6-30** | **≤5** |  |
| COPD |  |  |  |  |  |  |
| All | 1103/4928 | 1.00 | 1.52 (1.17-1.96) | 1.67 (1.31-2.12) | 2.21 (1.67-2.93) | <0.0001 |
| Male | 541/2512 | 1.00 | 1.42 (0.99-2.03) | 1.35 (0.96-1.90) | 1.99 (1.35-2.95) | 0.0023 |
| Female | 562/2416 | 1.00 | 1.61 (1.11-2.35) | 2.06 (1.46-2.92) | 2.48 (1.65-3.71) | <0.0001 |
| Emphysema |  |  |  |  |  |  |
| All | 850/5179 | 1.00 | 1.50 (1.12-2.01) | 1.73 (1.32-2.28) | 2.36 (1.73-3.23) | <0.0001 |
| Male | 464/2587 | 1.00 | 1.36 (0.93-2.01) | 1.41 (0.98-2.03) | 2.10 (1.39-3.19) | 0.0010 |
| Female | 386/2592 | 1.00 | 1.69 (1.08-2.64) | 2.25 (1.48-3.40) | 2.75 (1.71-4.44) | <0.0001 |
| Chronic bronchitis |  |  |  |  |  |  |
| All | 447/5550 | 1.00 | 2.04 (1.35-3.06) | 1.87 (1.26-2.77) | 2.98 (1.93-4.60) | <0.0001 |
| Male | 159/2879 | 1.00 | 2.33 (1.19-4.56) | 1.59 (0.82-3.09) | 2.99 (1.46-6.11) | 0.0231 |
| Female | 288/2671 | 1.00 | 1.84 (1.10-3.09) | 2.05 (1.26-3.34) | 2.99 (1.73-5.17) | 0.0001 |

COPD cases responded affirmatively to question(s) regarding diagnosis of emphysema (on study baseline questionnaire, follow-up questionnaire in 2006, or both), chronic bronchitis (on study baseline questionnaire), or both. ORs and 95% CIs determined using logistic regression with adjustments for age, gender (except in gender-stratified analyses), race, education, cigarettes/day, years smoked during lifetime, pack-years, age at smoking initiation, and lung cancer diagnosis prior to follow-up questionnaire. TTFC was categorical (>60 minutes as reference) with the exception of P-trend assessment where TTFC was treated as an ordinal variable. PLCO, Prostate, Lung, Colorectal, and Ovarian (PLCO) Cancer Screening Trial.

**Table D. Adjusted Odds Ratio (95% CI) for COPD according to TTFC among current smokers in PLCO, limited to participants with complete data on cigarettes per day at follow-up questionnaire**

|  | **No. Cases/**  **No. Controls** | **Time to First Cigarette (minutes)** | | | | **P-value for**  **Trend** |
| --- | --- | --- | --- | --- | --- | --- |
| **Disease Endpoint** |  | **>60 (Ref.)** | **31-60** | **6-30** | **≤5** |  |
| COPD |  |  |  |  |  |  |
| All | 1124/4907 | 1.00 | 1.45 (1.12-1.88) | 1.60 (1.26-2.04) | 2.14 (1.62-2.84) | <0.0001 |
| Male | 553/2488 | 1.00 | 1.40 (0.98-2.01) | 1.34 (0.95-1.88) | 1.99 (1.34-2.94) | 0.0022 |
| Female | 571/2419 | 1.00 | 1.49 (1.03-2.16) | 1.90 (1.35-2.67) | 2.27 (1.52-3.39) | <0.0001 |
| Emphysema |  |  |  |  |  |  |
| All | 806/4722 | 1.00 | 1.33 (0.99-1.80) | 1.48 (1.12-1.95) | 2.11 (1.52-2.91) | <0.0001 |
| Male | 443/2351 | 1.00 | 1.33 (0.90-1.98) | 1.30 (0.89-1.89) | 1.95 (1.27-3.01) | 0.0009 |
| Female | 363/2371 | 1.00 | 1.33 (0.84-2.10) | 1.73 (1.13-2.64) | 2.27 (1.39-3.70) | <0.0001 |
| Chronic bronchitis |  |  |  |  |  |  |
| All | 451/5542 | 1.00 | 2.00 (1.34-3.00) | 1.82 (1.23-2.68) | 2.96 (1.92-4.66) | <0.0001 |
| Male | 161/2864 | 1.00 | 2.30 (1.17-4.52) | 1.55 (0.80-3.02) | 3.04 (1.48-6.22) | 0.0189 |
| Female | 290/2678 | 1.00 | 1.80 (1.08-3.00) | 1.97 (1.22-3.20) | 2.91 (1.69-5.01) | 0.0002 |

COPD cases responded affirmatively to question(s) regarding diagnosis of emphysema (on study baseline questionnaire, follow-up questionnaire in 2006, or both), chronic bronchitis (on study baseline questionnaire), or both. ORs and 95% CIs determined using logistic regression with adjustments for age, gender (except in gender-stratified analyses), race, education, cigarettes/day, years smoked during lifetime, pack-years, age at smoking initiation, and lung cancer diagnosis prior to follow-up questionnaire. TTFC was categorical (>60 minutes as reference) with the exception of P-trend assessment where TTFC was treated as an ordinal variable. PLCO, Prostate, Lung, Colorectal, and Ovarian (PLCO) Cancer Screening Trial.

**Table E. Adjusted Odds Ratio (95% CI) for COPD according to TTFC among current smokers in PLCO, excluding participants missing** follow-up questionnaire data for any covariates

|  | **No. Cases/**  **No. Controls** | **Time to First Cigarette (minutes)** | | | | **P-value for**  **Trend** |
| --- | --- | --- | --- | --- | --- | --- |
| **Disease Endpoint** |  | **>60 (Ref.)** | **31-60** | **6-30** | **≤5** |  |
| COPD |  |  |  |  |  |  |
| All | 1036/4494 | 1.00 | 1.38 (1.06-1.80) | 1.45 (1.13-1.86) | 1.98 (1.48-2.65) | <0.0001 |
| Male | 516/2280 | 1.00 | 1.40 (0.97-2.03) | 1.23 (0.86-1.74) | 1.83 (1.22-2.75) | 0.0171 |
| Female | 520/2214 | 1.00 | 1.36 (0.92-2.00) | 1.72 (1.20-2.46) | 2.11 (1.39-3.22) | 0.0002 |
| Emphysema |  |  |  |  |  |  |
| All | 872/5157 | 1.00 | 1.43 (1.07-1.91) | 1.66 (1.27-2.18) | 2.28 (1.66-3.11) | <0.0001 |
| Male | 476 /2563 | 1.00 | 1.34 (0.91-1.98) | 1.40 (0.97-2.02) | 2.09 (1.38-3.18) | 0.0058 |
| Female | 396/2594 | 1.00 | 1.53 (0.99-2.36) | 2.03 (1.35-3.03) | 2.46 (1.54-3.94) | 0.0004 |
| Chronic bronchitis |  |  |  |  |  |  |
| All | 410/5090 | 1.00 | 2.00 (1.30-3.06) | 1.77 (1.17-2.67) | 2.82 (1.78-4.45) | 0.0001 |
| Male | 148/2634 | 1.00 | 2.56 (1.28-5.15) | 1.53 (0.76-3.08) | 3.05 (1.43-6.47) | 0.0010 |
| Female | 262/2456 | 1.00 | 1.67 (0.97-2.88) | 1.92 (1.15-3.20) | 2.68 (1.51-4.78) | 0.0427 |

COPD cases responded affirmatively to question(s) regarding diagnosis of emphysema (on study baseline questionnaire, follow-up questionnaire in 2006, or both), chronic bronchitis (on study baseline questionnaire), or both. ORs and 95% CIs determined using logistic regression with adjustments for age, gender (except in gender-stratified analyses), race, education, cigarettes/day, years smoked during lifetime, pack-years, age at smoking initiation, and lung cancer diagnosis prior to follow-up questionnaire. TTFC was categorical (>60 minutes as reference) with the exception of P-trend assessment where TTFC was treated as an ordinal variable. Gender not included as covariate in gender-stratified models. PLCO, Prostate, Lung, Colorectal, and Ovarian (PLCO) Cancer Screening Trial.

**Table F. Adjusted Odds Ratio (95% CI) for emphysema and chronic bronchitis among current smokers in PLCO, with mutual adjustment for emphysema/chronic bronchitis**

|  | **No. Cases/**  **No. Controls** | **Time to First Cigarette (minutes)** | | | | **P-value for**  **Trend** |
| --- | --- | --- | --- | --- | --- | --- |
| **Disease Endpoint** |  | **>60 (Ref.)** | **31-60** | **6-30** | **≤5** |  |
| Emphysema | 561/2533 | 1.00 | 1.3 (1.0-1.8) | 1.6 (1.2-2.1) | 2.0 (1.4-2.7) | <0.0001 |
| Chronic bronchitis | 575/2439 | 1.00 | 1.9 (1.3-2.9) | 1.6 (1.1-2.4) | 2.4 (1.6-3.8) | 0.001 |

Emphysema cases responded affirmatively to query about diagnosis of emphysema on the study baseline questionnaire, follow-up questionnaire in 2006, or both. Chronic bronchitis cases responded affirmatively to query at study baseline (1993-2001) about a doctor’s diagnosis of chronic bronchitis. ORs and 95% CIs determined using logistic regression with adjustments for age, gender, education, cigarettes/day, years smoked during lifetime, pack-years, age at smoking initiation, and lung cancer diagnosis prior to follow-up questionnaire. Models of emphysema risk were adjusted for chronic bronchitis (yes/no). Models of chronic bronchitis risk were adjusted for emphysema (yes/no). TTFC was categorical (>60 minutes as reference) with the exception of P-trend assessment where TTFC was treated as an ordinal variable. PLCO, Prostate, Lung, Colorectal, and Ovarian (PLCO) Cancer Screening Trial.

**Table G. Adjusted Odds Ratio (95% CI) for COPD among current smokers in PLCO, among Non-Hispanic Whites**

|  | **No. Cases/**  **No. Controls** | **Time to First Cigarette (minutes)** | | | | **P-value for**  **Trend** |
| --- | --- | --- | --- | --- | --- | --- |
| **Disease Endpoint** |  | **>60 (Ref.)** | **31-60** | **6-30** | **≤5** |  |
| COPD |  |  |  |  |  |  |
| All | 1007/4201 | 1.00 | 1.50 (1.14-1.97) | 1.61 (1.25-2.08) | 2.21 (1.64-2.98) | <0.0001 |
| Male | 505/2114 | 1.00 | 1.49 (1.03-2.16) | 1.27 (0.89-1.81) | 1.98 (1.31-2.99) | 0.0088 |
| Female | 502/2087 | 1.00 | 1.51 (1.01-2.25) | 2.04 (1.41-2.96) | 2.45 (1.58-3.78) | <0.0001 |
| Emphysema |  |  |  |  |  |  |
| All | 790/4418 | 1.00 | 1.47 (1.08-1.99) | 1.64 (1.23-2.18) | 2.33 (1.68-3.24) | <0.0001 |
| Male | 442/2177 | 1.00 | 1.48 (1.00-2.21) | 1.37 (0.94-2.00) | 2.14 (1.38-3.30) | 0.0031 |
| Female | 348/2241 | 1.00 | 1.44 (0.90-2.32) | 2.05 (1.33-3.18) | 2.58 (1.56-4.29) | <0.0001 |
| Chronic bronchitis |  |  |  |  |  |  |
| All | 399/4783 | 1.00 | 1.97 (1.28-3.03) | 1.84 (1.21-2.78) | 2.84 (1.78-4.51) | <0.0001 |
| Male | 142/2463 | 1.00 | 2.15 (1.08-4.26) | 1.32 (0.67-2.62) | 2.69 (1.28-5.66) | 0.0646 |
| Female | 257/2320 | 1.00 | 1.80 (1.03-3.14) | 2.18 (1.29-3.67) | 2.87 (1.59-5.19) | 0.0006 |

COPD cases responded affirmatively to question(s) regarding diagnosis of emphysema (on study baseline questionnaire, follow-up questionnaire in 2006, or both), chronic bronchitis (on study baseline questionnaire), or both. Race/ethnicity was self-reported. ORs and 95% CIs determined using logistic regression with adjustments for age, gender (except in gender-stratified analyses), education, cigarettes/day, years smoked during lifetime, pack-years, age at smoking initiation, and lung cancer diagnosis prior to follow-up questionnaire. TTFC was categorical (>60 minutes as reference) with the exception of P-trend assessment where TTFC was treated as an ordinal variable. PLCO, Prostate, Lung, Colorectal, and Ovarian (PLCO) Cancer Screening Trial.

**Table H. Adjusted Odds Ratio (95% CI) for COPD among current smokers in PLCO, among African Americans**

|  | **No. Cases/**  **No. Controls** | **Time to First Cigarette (minutes)** | | | | **P-value for**  **Trend** |
| --- | --- | --- | --- | --- | --- | --- |
| **Disease Endpoint** |  | **>60 (Ref.)** | **31-60** | **6-30** | **≤5** |  |
| COPD | 41/319 | 1.00 | 1.29 (0.34-4.86) | 1.77 (0.59-5.29) | 2.39 (0.71-8.02) | 0.1313 |
| Emphysema | 29/331 | 1.00 | 0.74 (0.16-3.43) | 1.14 (0.36-3.66) | 1.34 (0.36-4.98) | 0.5524 |

COPD cases responded affirmatively to question(s) regarding diagnosis of emphysema (on study baseline questionnaire, follow-up questionnaire in 2006, or both), chronic bronchitis (on study baseline questionnaire), or both. Chronic bronchitis is not shown separately due to small sample size. Race/ethnicity was self-reported. ORs and 95% CIs determined using logistic regression with adjustments for age, gender, education, cigarettes/day, years smoked during lifetime, pack-years, age at smoking initiation, and lung cancer diagnosis prior to follow-up questionnaire. TTFC was categorical (>60 minutes as reference) with the exception of P-trend assessment where TTFC was treated as an ordinal variable. PLCO, Prostate, Lung, Colorectal, and Ovarian (PLCO) Cancer Screening Trial.
